# Role of TLR4-Mediated PI3K/AKT/GSK-3*β* Signaling Pathway in Apoptosis of Rat Hepatocytes

**DOI:** 10.1155/2015/631326

**Published:** 2015-12-07

**Authors:** Xian Zhang, Daorong Jiang, Wei Jiang, Min Zhao, Jianhe Gan

**Affiliations:** ^1^Department of Infectious Disease, The First Affiliated Hospital of Soochow University, Suzhou, Jiangsu 215006, China; ^2^Department of Infectious Disease, Affiliated Hospital of Nantong University, Nantong, Jiangsu 226001, China; ^3^Department of Science Technology and Industry, Nantong University, Nantong, Jiangsu 226019, China; ^4^Department of Pathophysiology, Nantong University, Nantong, Jiangsu 226001, China

## Abstract

We investigated the mechanism of the Toll-like receptor 4- (TLR4-) mediated PI3K/AKT/GSK-3*β* signaling pathway in rat hepatocytes apoptosis induced by LPS. The cultured rat hepatocytes were treated with LPS alone or first pretreated with TLR4 inhibitor, AKT inhibitor, and GSK-3*β* inhibitor, respectively, and then stimulated with the same dose of LPS. Cell viability, cell apoptotic rate, and apoptosis morphology were assessed; the level of P-AKT^Ser473^, P-GSK-3β^Ser9^, and active Caspase-3 and the ratio of Bax/Bcl-2 were evaluated. The results indicated that cell viability decreased, while cell apoptotic rate increased with time after LPS stimulation. The expression of P-AKT^Ser473^ and P-GSK-3β^Ser9^ in the LPS group decreased compared with the control, while the level of active Caspase-3 and the ratio of Bax/Bcl-2 were significantly increased. These effects were attenuated by pretreatment with CLI-095. In addition, the apoptotic ratio decreased after pretreatment with LiCl but increased following pretreatment with LY294002. The expression of P-AKT^Ser473^ further decreased following pretreatment with LY294002 and the expression of P-GSK-3β^Ser9^ increased following pretreatment with LiCl. Moreover, pretreatment with CLI-095 weakened LPS-induced nuclear translocation of GSK-3*β*. Our findings suggest that the TLR4-mediated PI3K/AKT/GSK-3*β* signaling pathway is present in rat hepatocytes and participates in apoptosis of BRL-3A cells.

## 1. Introduction

Acute liver failure (ALF) has a rapid onset, low cure rate, and high mortality rate. The main pathological change is significant liver cell death which causes severe impairment of liver function [1]. Studies [2–4] have shown that apoptosis is one of the main forms of liver cell death in ALF. Apoptosis plays a very important role in the process of ALF. However, to date, the mechanism of cell apoptosis in ALF is unclear. The recently discovered toll-like receptors (TLRs), which are members of the pattern recognition receptor family, are attracting increasing attention due to their role in many infectious diseases and inflammatory lesions caused by nonpathogenic microorganisms. To date, 11 (TLR1–TLR11) toll-like receptors in this family have been identified, with different subtypes identifying the same pathogen-associated molecular patterns (PAMPs) shared by different microbes. TLR4, the first TLR-related protein to be discovered, identifies the cell wall component lipopolysaccharide (LPS) in Gram-negative bacteria. It was recently found that not only exogenous factors but also endogenous ligands such as heat shock protein can activate TLR4 [5, 6]. Takayashiki et al. [7, 8] showed that the liver cell membrane expressed TLR4, and the level increased significantly in mice with hepatic failure [9]. However, to date, there are no reports on whether TLR4-mediated signaling participates in liver cell apoptosis in ALF.

Among the signaling pathways related to cell apoptosis, the phosphatidylinositol 3-kinase- (PI3K-) serine/threonine kinase (AKT) signaling pathway is currently considered to be important in cell survival. This pathway mediates a variety of biological effects to inhibit apoptosis [10, 11]. Activated AKT exerts a wide range of biological effects by facilitating the phosphorylation of downstream substrates such as glycogen synthase kinase-3*β* (GSK-3*β*). The role of the TLR4-mediated PI3K/AKT/GSK-3*β* signaling pathway in liver cell apoptosis in ALF is unclear.

In this study, different drugs were used to weaken or strengthen the effect of the TLR4 signaling pathway. CCK-8 assay, immunofluorescence, Annexin V/PI, RT-PCR, and Western blotting technology were used to determine whether TLR4-mediated PI3K/AKT/GSK-3*β* signaling pathway participates in liver cell apoptosis so as to evaluate the role of the TLR4-mediated PI3K/AKT/GSK-3*β* signaling pathway in liver cell apoptosis in ALF. This study not only provides a theoretical basis for the prevention and treatment of ALF by regulating the apoptosis of liver cells but also provides a new target in the treatment of liver failure.

## 2. Material and Methods

### 2.1. Reagents and Antibodies

RPMI-1640 medium was purchased from Thermo Fisher (Shanghai, China). CCK-8 and Hoechst 33342 solution were obtained from Dojindo Laboratories (Tokyo, Japan). LPS, LY294002, and LiCl were obtained from Sigma-Aldrich (St. Louis, MO, USA). Annexin V-FITC/Propidium Iodide were obtained from Biouniquer Technology Co., Ltd. Antibodies of AKT, phospho-AKT, GSK-3*β*, P-GSK-3*β*, Bax, Bcl-2, and active caspase-3 were obtained from Cell Signaling Technology (Beverly, MA, USA). CLI-095 was obtained from Invivogen Biotechnology (San Diego, CA, USA). RNAiso Plus, SYBR Green Premix Ex Taq II, and PrimeScript RT Reagent Kit (Perfect Real Time) were purchased from TaKaRa (Takara Bio Inc., Shiga, Japan). PCR primers were synthesized at RQ Biotech (Shanghai, China).

### 2.2. Cell Culture and Treatment

The rat liver cell line, BRL-3A, was purchased from Chinese Academy of Sciences (Shanghai, China). BRL-3A cells were maintained in RPMI-1640 medium supplemented with 10% fetal calf serum, penicillin (100 U/mL), and streptomycin (100 *μ*g/mL) in a humidified incubator (37°C, 5% CO_2_). BRL-3A cells grown to 80–90% confluence in complete growth medium were used in the experiments. The cells were treated with LPS (10 *μ*g/mL) for 1, 3, 6, 12, and 24 h.

### 2.3. Measurement of Cell Viability at Different Time Points

Cell viability was determined using the CCK-8 assay. Cells were seeded at a density of 1 × 10^4^ cells/mL in 96-well polystyrene culture plates. After LPS stimulation, the medium was removed and replaced with fresh medium CCK-8. After 2 h of incubation, absorbance was measured at 450 nm using a microplate reader (SpectraMax 250, Sunnyvale, CA, USA).

### 2.4. Apoptotic Rates Analyzed by Flow Cytometric Analysis Using Annexin V and Propidium Iodide

Cells were seeded in six-well plates at 2 × 10^5^ cells/well. The cells were washed twice with PBS, treated with trypsin, and stained with Annexin V-FITC and Propidium Iodide in binding buffer. Ten thousand events were collected from each sample. The stained cells were analyzed within 1 h using flow cytometry (BD Biosciences, San Jose, CA, USA).

### 2.5. Experimental Groups

The cells were divided into five groups. The treatments were as follows: the normal control group received no treatment; BRL-3A cells in the LPS group were treated with LPS (10 *μ*g/mL) for 24 h; the CLI-095 + LPS group, the LY294002 + LPS group, and the LiCl + LPS group were pretreated with CLI-095 (1 *μ*g/mL), LY294002 (50 *μ*mol/L), and LiCl (10 *μ*mol/L) for 2 h, respectively, and then stimulated with some dose of LPS. The representative data shown in this paper were reproducible in three independent experiments.

### 2.6. Western Blotting Analysis

Western blotting was performed to determine the expression of AKT, P-AKT^Ser473^, GSK-3*β*, P-GSK-3*β* ^Ser9^, active caspase-3, Bcl-2, and Bax. Cells from the different groups were collected and lysed in RIPA buffer with 1x cocktail protease inhibitor. Samples were separated on a 12% sodium dodecyl sulfate-polyacrylamide gel and transferred to polyvinylidene difluoride (PVDF) membranes. The membranes were first blocked at room temperature with 5% skimmed milk in 1x TBST buffer for 2 h and then incubated with primary antibody (1 : 1000) overnight at 4°C. PVDF membranes were washed with 1x TBST three times, followed by incubation with horseradish peroxidase conjugated anti-rabbit IgG (1 : 10000) for 2 h. After washing in 1x TBST three times, the membranes were visualized by the ECL system (Millipore, New York, NY, USA).

### 2.7. Nuclear Translocation of GSK-3*β* in BRL-3A Cells Was Determined by the Double Labeling Immunofluorescence Assay

BRL-3A cells were incubated and each group was treated as above. The round glass slides were taken out and placed in a new plate and then washed once with PBS and fixed in 4% paraformaldehyde for 30 min. After washing with PBS three times, the cell membrane was permeabilized with 0.3% Triton X-100 for 20 min and then blocked with 3% bovine serum albumin for 20 min. For the detection of GSK-3*β*, the coverslips were incubated with rabbit anti-GSK-3*β* polyclonal antibody (1 : 100; Cell Signaling) at 4°C overnight. After washing three times with PBS, the cells were then incubated with Alexa Fluor 488-conjugated goat anti-rabbit IgG secondary antibody (1 : 1200, Invitrogen, New York, NY, USA) for 1 h and then washed with PBS. Nuclei were then stained with Hoechst 33342 solution (1 : 1000) for 15 min. After washing twice with ddH_2_O, the coverslips were observed under a fluorescence microscope (BX50WI, Olympus, Tokyo, Japan).

### 2.8. The Expression of Caspase-3 and Bax/Bcl-2 mRNA Using Real-Time Quantitative PCR

Total RNA was extracted from treated BRL-3A cells using TRIzol method (Invitrogen, Carlsbad, CA, USA). Total RNA was subsequently reverse-transcribed into cDNA following the reverse transcription protocol. Gene expression levels were measured by real-time PCR on ABI7300 machine (Applied Biosystems, CA, USA). The sequences of primers were as follows:

 R-Casp3-F: 5′GGACTGCGGTATTGAGACAGAC 3′.
 R-Casp3-R: 5′CCTTCCGGTTAACACGAGTGA 3′.
 R-Bax-F: 5′CACCAAGAAGCTGAGCGAGT 3′.
 R-Bax-R: 5′AAGTTGCCGTCTGCAAACAT 3′.
 R-Bcl-2-F: 5′TGGTGGACAACATCGCTCT 3′.
 R-Bcl-2-R: 5′CAGCCAGGAGAAATCAAACAG 3′.
 R-beta-actin-F: 5′AGTGTGACGTTGACATCCGTAA 3′.
 R-beta-actin-R: 5′GGACAGTGAGGCCAGGATAGA 3′.

### 2.9. Statistical Analysis

All data were analyzed with SPSS 17.0 software (SPSS Inc., Chicago, IL, USA). Data were expressed as mean ± SEM. Statistical significance of differences between groups was determined by one-way analysis of variance followed by Tukey's post hoc multiple comparison tests. *P* values < 0.05 were considered statistically significant.

## 3. Results

### 3.1. Effect of LPS on Cell Viability, Apoptotic Rate, and Morphology of BRL-3A Cells

**Figure 1:**
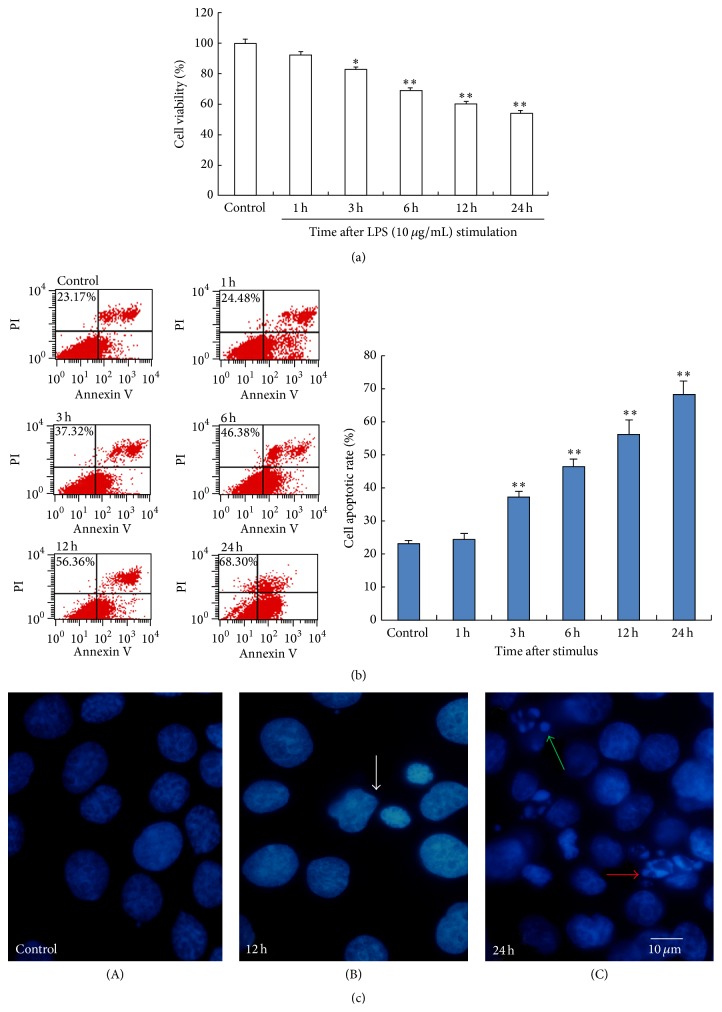
The effect of LPS on BRL-3A cells. The control group was untreated, while LPS (10 *μ*g/mL) was used to treat separate groups of BRL-3A cells. (a) Cell viability of BRL-3A cells at 0–24 h after LPS stimulation was detected by CCK-8 assay. (b) Cells were stained for FITC-Annexin V and Propidium Iodide and then sorted and analyzed quantitatively by flow cytometry. Cell apoptotic rates are reported in the histograms. (c) Hoechst 33342 fluorescence staining of BRL-3A cells at different time points after LPS stimulation. (A) Control group: intact BRL-3A cells. (B) Karyopyknosis (white arrow) 12 h after LPS stimulation. (C) Nuclear fragmentation (red arrow) and apoptotic body (green arrow) 24 h after stimulation. Scale bars = 10 *μ*m. Data were mean ± SEM. ^*∗*^ *P* < 0.05 and ^*∗∗*^ *P* < 0.01 versus control group.

The results of CCK-8 assay indicated that, compared with the control group, cell viability decreased with time after LPS stimulation. After being stimulated for 24 h, the activity of BRL-3A cells descended to 54% (*P* < 0.01) (Figure 1(a)). In contrast to cell viability, cell apoptotic rate increased with time after LPS stimulation. The highest percentage of apoptosis appeared at 24 h (*P* < 0.05), and the percentage of necrotic cells also peaked at 24 h (Figure 1(b)). Under the fluorescent microscope, cells in normal control group were intact. Karyopyknosis can be seen 12 h after LPS simulation; nuclear fragmentation and apoptotic bodies were visible 24 h after simulation of LPS (Figure 1(c)).

### 3.2. TLR4 Participated in Cell Apoptosis Caused by LPS

**Figure 2:**
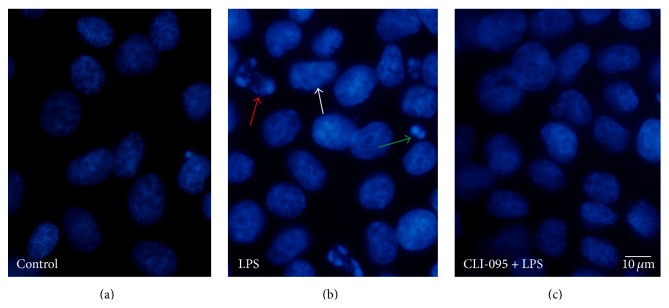
The effect of TLR4 inhibitor on apoptosis caused by LPS using Hoechst33342 fluorescence staining. (a) Control group: intact BRL-3A cells. (b) Apoptotic body (green arrow), nuclear fragmentation (red arrow), and karyopyknosis (white arrow) 24 h after LPS simulation. (c) CLI-095 + LPS group was pretreated with CLI-095 for 2 h and then treated with some dose of LPS for 24 h. Apoptotic body and nuclear fragmentation cannot be seen. Scale bars = 10 *μ*m.

We observed the morphological changes of apoptosis in BRL-3A cells using Hoechst 33342 staining after LPS stimulation with or without CLI-095 pretreatment. The nuclei showed a two-tone structure in the normal control group. In the LPS group, apoptotic nuclei were enhanced and deformed to cylindrical or pyknotic crumb structure. In the CLI-095 + LPS group, apoptotic cells were significantly reduced, with no visible apoptotic bodies (Figure 2).

### 3.3. Inhibition of the TLR4/PI3K/AKT/GSK-3*β* Signaling Pathway Affected BRL-3A Cells Apoptosis

**Figure 3:**
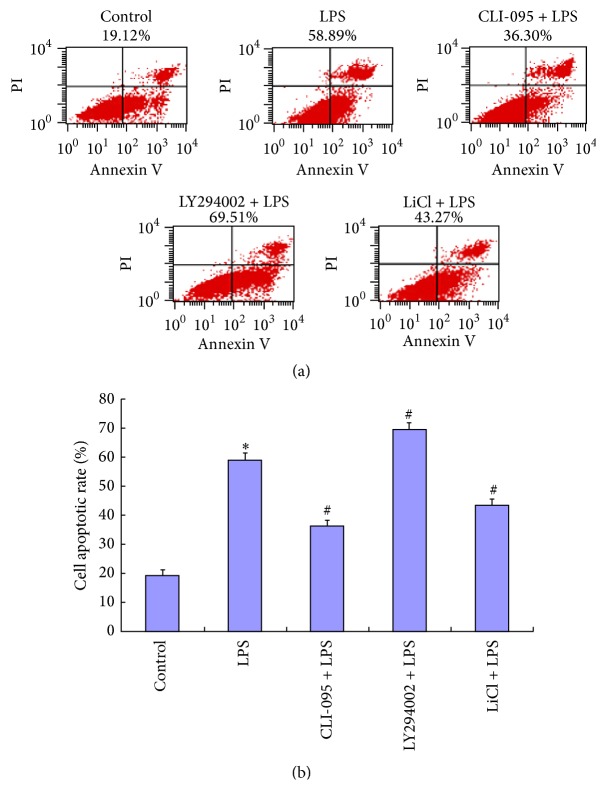
Apoptotic rates of BRL-3A cells in different groups detected by flow cytometry. The control group was untreated. The LPS group was treated with LPS (10 *μ*g/mL) for 24 h. CLI-095 + LPS group was pretreated with CLI-095 for 2 h and then treated with some dose of LPS for 24 h. The LY294002 + LPS group was pretreated with LY294002 for 2 h and then treated with LPS for 24 h. The LiCl + LPS group was pretreated with LiCl for 2 h and then treated with LPS for 24 h. (a) Cells were stained for FITC-Annexin V and Propidium Iodide and then sorted and analyzed quantitatively by flow cytometry. (b) Cell apoptotic rates are reported in the histograms. Data were mean ± SEM. ^*∗*^ *P* < 0.05 versus control group. ^#^ *P* < 0.05 versus LPS group.

According to the results of flow cytometry, the apoptotic rate of BRL-3A cells stimulated by LPS was significantly higher than that in the control group. Compared with LPS group, the apoptotic rate of BRL-3A cells decreased if pretreated with CLI-095 or GSK-3*β* inhibitor but increased if pretreated with LY294002 (Figure 3).

### 3.4. Effects of TLR4/PI3K/AKT/GSK-3*β* Signaling Pathway Inhibition on the Protein Expression of AKT, P-AKT^Ser473^, GSK-3*β*, and P-GSK-3*β* ^Ser9^ in BRL-3A Cells

**Figure 4:**
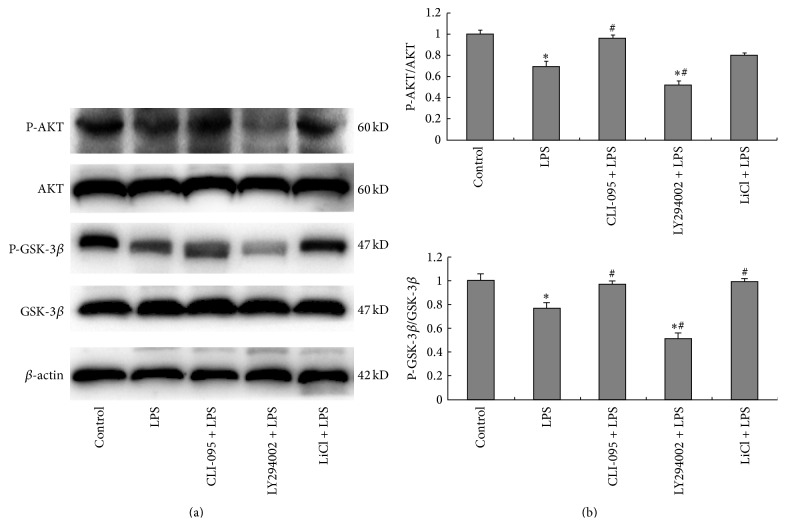
Comparison of protein kinase B (AKT) and glycogen synthase kinase-3*β* (GSK-3*β*) phosphorylation levels among different groups. The five groups were treated as above 3. (a) Representative Western blot analysis of AKT, P-AKT^Ser473^, GSK-3*β*, and P-GSK-3*β* ^Ser9^ protein of BRL-3A cells stimulated by LPS. The total AKT and GSK-3*β* were used as a control. (b) Quantitative analysis of P-AKT^Ser473^/AKT and P-GSK-3*β* ^Ser9^/GSK-3*β*. Data were mean ± SEM (*n* = 3). ^*∗*^ *P* < 0.05 versus control group. ^#^ *P* < 0.05 versus LPS group.

Western blotting revealed that the expression of both P-AKT^Ser473^ and P-GSK-3*β* ^Ser9^ in BRL-3A cells stimulated by LPS decreased compared with the control group (*P* < 0.05). Compared with the LPS group, the expression of P-AKT^Ser473^ and P-GSK-3*β* ^Ser9^ increased in CLI-095 + LPS group (*P* < 0.05), the expression of P-AKT^Ser473^ further decreased in LY294002 + LPS group (*P* < 0.05), and the expression of P-GSK-3*β* ^Ser9^ increased in LiCl + LPS group (*P* < 0.05) (Figure 4).

### 3.5. Effect of TLR4 Inhibition, GSK-3*β* Inhibition, and AKT Inhibition on GSK-3*β* Nuclear Translocation Induced by LPS

**Figure 5:**
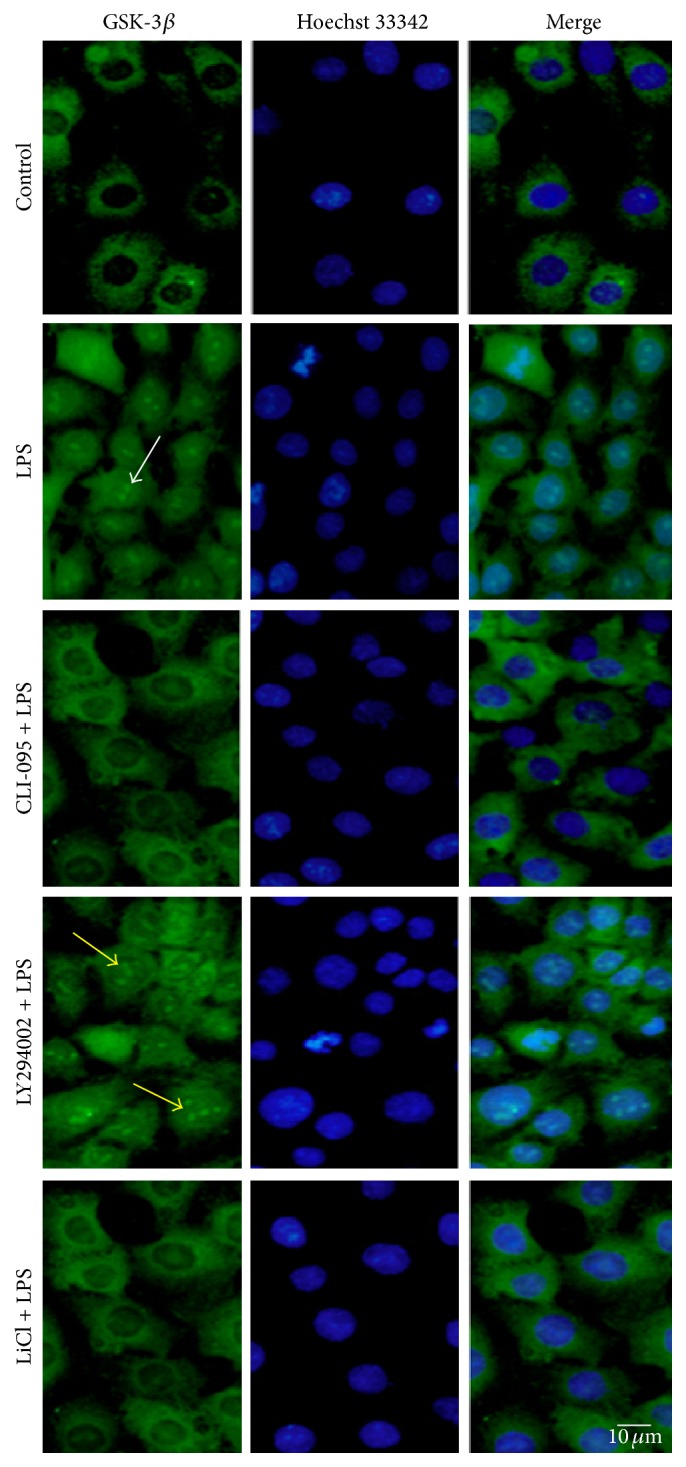
TLR4 inhibition, GSK-3*β* inhibition, and AKT inhibition affect GSK-3*β* translocation induced by LPS. The five groups were treated as above 4. GSK-3*β* translocated to the nucleus in LPS group (white arrow) and LY294002 + LPS group (yellow arrows). BRL-3A cells were incubated with GSK-3*β* antibody. Nucleus was visualized using Hoechst 33342 (blue). Immunofluorescence microscopy was used to detect the location of GSK-3*β* (green). Scale bars = 10 *μ*m.

Immunofluorescence technique was adopted to observe the nuclear translocation of GSK-3*β*. GSK-3*β* stained green and was located in the cytoplasm in control group. After 24 h of LPS stimulation, the majority of GSK-3*β* was translocated to the nucleus. However, in CLI-095 + LPS group, the nuclear translocation was obviously reduced. The LY294002 + LPS group showed a more obvious translocation, whereas the LiCl + LPS group showed lighter translocation compared with LPS group (Figure 5).

### 3.6. Effects of TLR4/PI3K/AKT/GSK-3*β* Signaling Pathway Inhibition on the Production of Bax, Bcl-2, and Active Caspase-3 after LPS Stimulation in BRL-3A Cells

**Figure 6:**
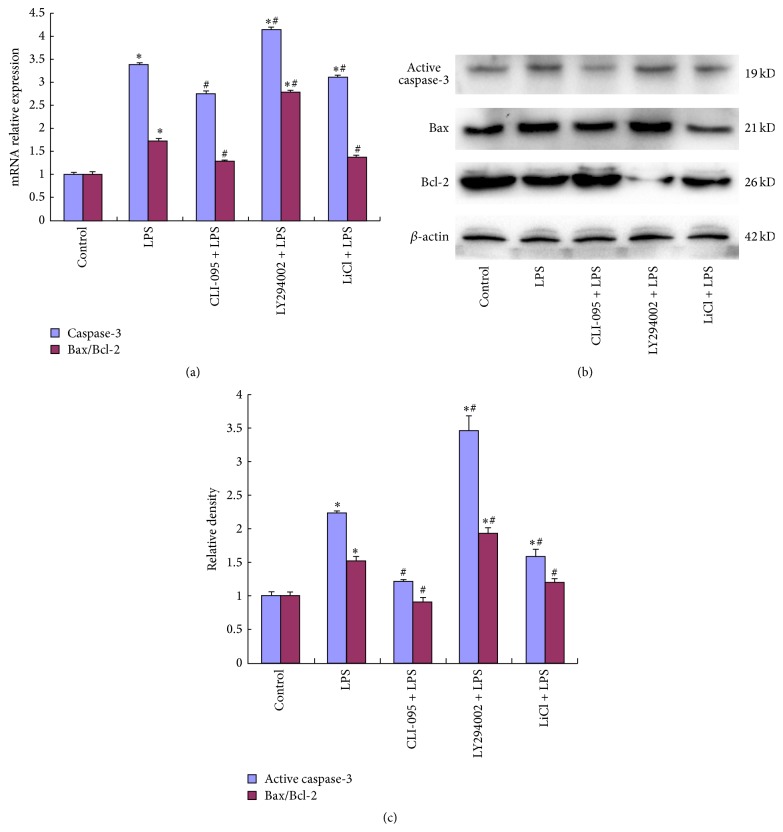
TLR4/PI3K/AKT/GSK-3*β* signaling pathway inhibition affects production of Bax, Bcl-2, and active caspase-3. The five groups were treated as above 4. (a) The mRNA expression of Bax/Bcl-2 and caspase-3 was detected by RT-qPCR. (b) Quantification (relative density) of the intensity of staining of active caspase-3, Bax, and Bcl-2 protein detected by Western blot. (c) Representative Western blot of active caspase-3, Bax, and Bcl-2 protein from BRL-3A cells. *β*-actin was used as an internal control. Data were mean ± SEM (*n* = 3). ^*∗*^ *P* < 0.05 versus control group. ^#^ *P* < 0.05 versus LPS group.

To further examine the expression profiles of Bax, Bcl-2, and active caspase-3, RT-qPCR was adopted to detect the mRNA expression of Bax, Bcl-2, and caspase-3. The level of caspase-3 and the ratio of Bax/Bcl-2 in BRL-3A cells stimulated by LPS were significantly increased compared with the control group (*P* < 0.05). The effect attenuated by pretreatment with CLI-095 and LiCl and strengthened by pretreatment with LY294002 (Figure 6(a)). The results of Western blotting were in accordance with the results of RT-qPCR (Figures 6(b) and 6(c)).

## 4. Discussion

Studies in recent years have shown that liver cell apoptosis plays an important role in the pathological process of ALF [12–15]. Riordan and Williams [16] reported that apoptosis was a major pathological morphological feature of ALF. Obvious apoptosis has been found in liver injury caused by factors such as virus and bacterial endotoxin [2, 17–20]. Liver cell apoptosis is the most important molecular mechanism in hepatic failure [21, 22]. A study on the mechanism of apoptosis can reveal the process of acute liver injury and liver failure caused by endotoxin. LPS is the main component of cell walls in Gram-negative bacilli. Mainly, by binding corresponding receptors on the cell membrane, LPS can initiate intracellular signals, activate nuclear transcription factor kappa B (NF-*κ*B) and the protein kinase of P38 lightning mitogen, start gene transcription, and induce releasing many types of inflammatory factors and cellular toxic substances including TNF-a, IL-1*β*, NO, and superoxide, thus exerting its toxic effects. These inflammatory factors, especially TNF-a, can induce apoptosis and necrosis of liver cells [23]. By increasing the expression of CD14, TNF-related apoptosis inducing ligand, and TLR4, LPS can induce a systemic inflammatory response and lead to multiple organ damage [24]. In recent years, following further research on ALF, a mechanism for apoptosis has been put forward. Scholars successfully replicated apoptosis in the hepatic cell line (BRL-3A) and in animals with liver failure using LPS. They found that LPS had an obvious damaging effect on the BRL-3A liver cell line, which was manifested as a significant decrease in cell viability and a significant increase in cell apoptosis after LPS stimulation [25, 26]. In this study, markedly reduced cell viability revealed that LPS had a damaging effect on BRL-3A cells. Under the fluorescence microscope, karyopyknosis was seen 12 h after LPS stimulation and pieces of nuclei were visible after 24 h. The percentage of apoptosis increased in a time-dependent manner. LPS had a direct toxic effect on cultured BRL-3A cells and led to apoptosis.

The current studies focused on TLR4, a member of the TLRs family. As a membrane receptor protein, TLR4 mainly recognizes peptidoglycan in Gram-positive bacteria, lipopolysaccharide in Gram-negative bacteria, and heat shock protein. Studies have confirmed that the expression of TLR4 is increased in liver injury and ALF [9]. Under fluorescent microscopy, compared with the LPS group, apoptotic cells were significantly reduced in CLI-095 + LPS group. Quantitative analysis showed that LPS-induced apoptosis was effectively reduced after pretreatment with CLI-095. The results provide evidence for the fact that TLR4 participates in BRL-3A cell apoptosis caused by LPS.

As described above, TLR4 was involved in BRL-3A cell apoptosis caused by LPS. However, it has not been fully elucidated whether the TLR4-mediated PI3K/AKT/GSK-3*β* coupling signaling pathway is involved in BRL-3A cell apoptosis and its signal transduction mechanism. Phosphatidylinositol 3-kinase (PI3K) is an important member of the phospholipid kinase family [27, 28]. Of many PI3K-mediated signaling pathways, the PI3K/AKT signaling pathway is particularly important in regulating apoptosis [29]. Activated PI3K can promote formation of the second messenger, PIP3, which activates AKT through phosphorylation. AKT, also known as serine/threonine protein kinase B (PKB), is an important downstream molecule and a direct downstream target of PI3K. Activated AKT activates or inhibits its downstream substrates to regulate cell proliferation, differentiation, apoptosis, and other important processes [30]. It exerts a wide range of biological effects mainly by promoting the phosphorylation of Bax (one of the apoptosis promoters of the Bcl-2 family), mTOR (mammalian target of rapamycin), glycogen synthase kinase-3 (GSK-3), and other downstream substrates [31]. Cell apoptosis is the result of waterfall gene expression, and many gene products are involved in the occurrence and regulation of apoptosis. The PI3K/AKT signaling pathway participates in apoptosis by regulating apoptosis-related genes. P-AKT inactivates GSK-3*β* by phosphorylating it at Ser9, thus resisting apoptosis. GSK-3*β* plays a vital regulatory role in various activities such as cell growth, differentiation, apoptosis, and signal transduction. GSK-3*β* also induces apoptosis through phosphorylating Bax, which then enters the mitochondria and induces the release of cytochrome C to the cytoplasm.

We investigated whether the TLR4/PI3K/AKT/GSK-3*β* signaling pathway participates in apoptosis of BRL-3A cells. The PI3K-AKT inhibitor, LY294002, was used to negatively regulate this pathway in order to determine the influence of PI3K-AKT on BRL-3A cell apoptosis. LY294002 is a widely used specific PI3K inhibitor. It can inactivate the pathway by inhibiting the catalytic activity of PI3Kp110 subunits, thus blocking the production of the downstream substrate PIP3 [32, 33]. Ser473 is a major phosphorylation site of AKT [34]. Lithium chloride (LiCl) is a well-known and relatively specific inhibitor of GSK-3*β*. Lithium can promote the phosphorylation of GSK-3*β* at serine 9 residues and induce P-GSK-3*β* ^Ser9^. P-GSK-3*β* ^Ser9^ is the inactivated form of GSK-3*β*, the content of which represents the activity of GSK-3*β*. P-AKT inhibits the activity of GSK-3*β*-mediated apoptosis through the phosphorylation of GSK-3*β*.

According to the results of flow cytometry and Western blotting, the apoptotic rate in the LPS group increased significantly compared with the control group. Low AKT activity was detected based on decreased expression of P-AKT^Ser473^ and high GSK-3*β* activity was detected based on decreased expression of P-GSK-3*β* ^Ser9^ in the LPS group. LPS-induced apoptosis was effectively reduced after pretreatment with TLR4 inhibitor CLI-095. Our results indicated that LPS participated in BRL-3A cell apoptosis through the activation of TLR4. Activated TLR4 reduced P-AKT^Ser473^ and P-GSK-3*β* ^Ser9^ expression in BRL-3A cells. This effect was weakened by pretreatment with the TLR4 inhibitor. Stimulated by the same dose of LPS after inactivating AKT (pretreatment with LY294002), the expression level of P-AKT^Ser473^ was lower than that in LPS group. When P-GSK-3*β* activity was inhibited (pretreatment with LiCl) and then stimulated with same dose of LPS, the expression level of P-GSK-3*β* ^Ser9^ was higher than that in LPS group. These results showed that the TLR4/PI3K/AKT/P-GSK-3*β* signaling pathway is involved in BRL-3A cell apoptosis.

Recent studies have shown that GSK-3*β* regulates cell differentiation, proliferation, survival, and apoptosis by affecting many signals, structural proteins, and transcription factors. More researchers are attaching great importance to GSK-3*β* as a target for the treatment of liver failure [35], neurodegenerative diseases, and other diseases. GSK-3*β* participates in the phosphorylation of more than 50 downstream substrates, such as *β* catenin (critical signaling protein of the Wnt signaling pathway), elF2B (the key factor in protein translation), and tau protein (the main protein related to stability of microtubules), thus regulating a variety of physiological processes [10]. Under normal conditions, GSK-3*β* concentrates in the cytoplasm mainly in the phosphorylated form (P-GSK-3*β* ^ser9^). When activated by a variety of apoptotic signals, active GSK-3*β* translocates from the cytoplasm to the nucleus and then participates in the development of apoptosis activities. In this study, we found that GSK-3*β* translocated to the nucleus in LPS group and LY294002 + LPS group. The effect of GSK-3*β* nuclear translocation was significantly weakened in CLI-095 + LPS group and LiCl + LPS group. Activation of TLR4 or inhibition of PI3K/AKT can activate GSK-3*β* and vice versa. These results suggest that activation or inhibition of the TLR4/PI3K/AKT/GSK-3*β* signaling pathway affects the activation of GSK-3*β* through nuclear translocation.

PI3K/AKT can activate many antiapoptotic genes (such as Bcl-2 and Bcl-XL) and inhibit a series of proapoptotic genes (such as Bax, caspase, and p53) [36, 37]. Bcl-2 family, caspases, and p53 belong to the downstream substrates of PI3K/AKT signaling pathway. The Bcl-2 family, an apoptosis-related protein gene family [38, 39], can be divided into two categories: antiapoptotic genes and proapoptotic genes. The former include Bcl-2, Bcl-XL, and Bcl-w. The latter include Bax, Bak, and Bok. Bcl-2 inhibits apoptosis by forming dimers with Bax after inactivating it. There is a balance between Bax and Bcl-2. An increase in Bax hastens apoptosis, while too much Bcl-2 inhibits apoptosis. The ratio of Bax/Bcl-2 in apoptosis plays a key role in liver failure. Caspases family is the executor in the process of cell apoptosis. Cysteine-containing aspartic-specific protease-3 (caspase-3), the key enzyme in cell apoptosis, is located downstream of a series of cascades [40]. It is generally accepted that apoptosis is the result of a series of highly regulated caspase cascades. Caspase-3 plays a dominant role in these cascades. Activated caspase-3 specifically cuts DNA and inactivates the related protease in DNA damage repair, resulting in apoptosis [41]. p53 is one of the important apoptosis inducing genes in the body. p53 induced apoptosis via regulating the gene expression of Bc1-2 and Bax. p53 protein, which is a direct Bax activator, can specifically inhibit the expression of Bc1-2. The activation of p53 as a transcription factor can increase the expression of proapoptotic genes and promote the transcription of Bax [42–44]. Overexpression of p53 can induce apoptosis. In contrast, inhibition of p53 can resist cell apoptosis and promote cell growth [44, 45]. It is indicated that AKT can inhibit cell apoptosis via phosphorylation of Bax, p53, and caspases after activation of PI3K/AKT pathway. PI3K/AKT signal pathway inhibits apoptosis and protects cell survival through enhancing the antiapoptotic role of the Bcl-2 family members (such as Bcl-2 and Bcl-XL), inhibiting the expression of proapoptotic genes, inhibiting caspase activation, preventing the release of apoptosis factor from mitochondria, and promoting the formation of inhibitor of apoptosis (IAP) proteins [46–48].

In order to study further whether TLR4/PI3K/AKT/GSK-3*β* pathway participates in mitochondrial apoptosis in BRL-3A cells, we detected the expression of Bax, Bcl-2, and active caspase-3 in each group. The results demonstrated that after activation of TLR4 by LPS the Bax/Bcl-2 ratio and expression of active caspase-3 were increased, suggesting that factors promoting cellular apoptosis were dominant, thus further activating caspase-3 and inducing a mitochondrial apoptosis pathway. After TLR4 inhibition, the expression of Bax/Bcl-2 and active caspase-3 obviously decreased. Inhibiting the activity of AKT by PI3K/AKT inhibitor (LY294002) increased the expression of Bax/Bcl-2 and active caspase-3, which can be cut down by inhibiting GSK-3*β* activity using GSK-3*β* inhibitor (LiCl). This indicated that LPS can regulate the Bax/Bcl-2 ratio and activates caspase-3 expression by activating the TLR4/PI3K/AKT/GSK-3*β* signaling pathway to promote apoptosis.

## 5. Conclusion

The TLR4-mediated PI3K/AKT/GSK-3*β* signaling pathway is present in BRL-3A cells. After activation of TLR4, expression of P-AKT^Ser473^ and P-GSK-3*β* ^Ser9^ decreases, and the Bax/Bcl-2 ratio and activated caspase-3 level increase; thus the rate of cell apoptosis increases. During this process, TLR4/PI3K/AKT/GSK-3*β* signaling pathway participates in the regulation of cell apoptosis. This study provides a new mechanism for liver cell apoptosis and a target for the treatment of liver failure.

## References

[1] Shirozu K., Tokuda K., Marutani E., Lefer D., Wang R., Ichinose F. (2014). Cystathionine *γ*-Lyase deficiency protects mice from Galactosamine/lipopolysaccharide-induced acute liver failure. *Antioxidants and Redox Signaling*.

[2] Kasahara I., Saitoh K., Nakamura K. (2000). Apoptosis in acute hepatic failure: histopathological study of human liver tissue using the tunel method and immunohistochemistry. *Journal of Medical and Dental Sciences*.

[3] Liu D. X. (2001). A new hypothesis of pathogenetic mechanism of viral hepatitis B and C. *Medical Hypotheses*.

[4] Cheng Y. J., Shyu W. C., Teng Y. H., Lan Y. H., Lee S. D. (2014). Antagonistic interaction between cordyceps sinensis and exercise on protection in fulminant hepatic failure. *The American Journal of Chinese Medicine*.

[5] Ohashi K., Burkart V., Flohé S., Kolb H. (2000). Cutting edge: heat shock protein 60 is a putative endogenous ligand of the toll-like receptor-4 complex. *Journal of Immunology*.

[6] Kim S. Y., Jeong E., Joung S. M., Lee J. Y. (2012). PI3K/Akt contributes to increased expression of Toll-like receptor 4 in macrophages exposed to hypoxic stress. *Biochemical and Biophysical Research Communications*.

[7] Takayashiki T., Yoshidome H., Kimura F. (2004). Increased expression of toll-like receptor 4 enhances endotoxin-induced hepatic failure in partially hepatectomized mice. *Journal of Hepatology*.

[8] Benias P. C., Gopal K., Bodenheimer H., Theise N. D. (2012). Hepatic expression of toll-like receptors 3, 4, and 9 in primary biliary cirrhosis and chronic hepatitis C. *Clinics and Research in Hepatology and Gastroenterology*.

[9] Cook D. N., Pisetsky D. S., Schwartz D. A. (2004). Toll-like receptors in the pathogenesis of human disease. *Nature Immunology*.

[10] Fulda S. (2013). Modulation of mitochondrial apoptosis by PI3K inhibitors. *Mitochondrion*.

[11] Johnson-Farley N. N., Patel K., Kim D., Cowen D. S. (2007). Interaction of FGF-2 with IGF-1 and BDNF in stimulating Akt, ERK, and neuronal survival in hippocampal cultures. *Brain Research*.

[12] Guicciardi M. E., Gores G. J. (2010). Apoptosis as a mechanism for liver disease progression. *Seminars in Liver Disease*.

[13] Wang K. (2014). Molecular mechanisms of liver injury: apoptosis or necrosis. *Experimental and Toxicologic Pathology*.

[14] Tagami A., Ohnishi H., Hughes R. D. (2003). Increased serum soluble Fas in patients with acute liver failure due to paracetamol overdose. *Hepato-Gastroenterology*.

[15] Kim S.-J., Kim K.-M., Park J., Kwak J.-H., Kim Y. S., Lee S.-M. (2013). Geniposidic acid protects against D-galactosamine and lipopolysaccharide-induced hepatic failure in mice. *Journal of Ethnopharmacology*.

[16] Riordan S. M., Williams R. (2003). Mechanisms of hepatocyte injury, multiorgan failure, and prognostic criteria in acute liver failure. *Seminars in Liver Disease*.

[17] Jaeschke H., Gujral J. S., Bajt M. L. (2004). Apoptosis and necrosis in liver disease. *Liver International*.

[18] Possamai L. A., McPhail M. J. W., Quaglia A. (2013). Character and temporal evolution of apoptosis in acetaminophen-induced acute liver failure. *Critical Care Medicine*.

[19] Doggrell S. A. (2004). Suramin: potential in acute liver failure. *Expert Opinion on Investigational Drugs*.

[20] Morio Y., Tsuji M., Inagaki M. (2013). Ethanol-induced apoptosis in human liver adenocarcinoma cells (SK-Hep1): Fas- and mitochondria-mediated pathways and interaction with MAPK signaling system. *Toxicology in Vitro*.

[21] Togo S., Kubota T., Matsuo K. (2004). Mechanism of liver failure after hepatectomy. *Nippon Geka Gakkai zasshi*.

[22] Eichhorst S. T. (2005). Modulation of apoptosis as a target for liver disease. *Expert Opinion on Therapeutic Targets*.

[23] Wang Y.-M., Feng G.-H., Huang F., Li Y., Zhao G.-Z. (2003). Tumor necrosis factor-alpha, caspase-3 expression and hepatocyte apoptosis in fulminanting hepatic failure. *Zhonghua Nei Ke Za Zhi*.

[24] Herzum I., Renz H. (2008). Inflammatory markers in SIRS, sepsis and septic shock. *Current Medicinal Chemistry*.

[25] Kim S.-J., Cho H.-I., Kim S.-J. (2014). Protective effect of linarin against d-galactosamine and lipopolysaccharide-induced fulminant hepatic failure. *European Journal of Pharmacology*.

[26] Su G. L. (2002). Lipopolysaccharides in liver injury: molecular mechanisms of Kupffer cell activation. *American Journal of Physiology—Gastrointestinal and Liver Physiology*.

[27] Aksamitiene E., Kiyatkin A., Kholodenko B. N. (2012). Cross-talk between mitogenic Ras/MAPK and survival PI3K/Akt pathways: a fine balance. *Biochemical Society Transactions*.

[28] Zhu M. Y., Guo J. L., Xia H. (2014). The anti-apoptotic effect of cytoplasmic alpha-fetoprotein in hepatoma cells induced by all-trans retinoic acid involves activation of the PI3K/AKT signaling pathway. *Chinese Journal of Hepatology*.

[29] Hanada M., Feng J., Hemmings B. A. (2004). Structure, regulation and function of PKB/AKT—a major therapeutic target. *Biochimica et Biophysica Acta—Proteins and Proteomics*.

[30] Atkins R. J., Dimou J., Paradiso L. (2012). Regulation of glycogen synthase kinase-3 beta (GSK-3*β*) by the Akt pathway in gliomas. *Journal of Clinical Neuroscience*.

[31] Yang L., Xie S., Jamaluddin M. S. (2005). Induction of androgen receptor expression by phosphatidylinositol 3-kinase/Akt downstream substrate, FOXO3a, and their roles in apoptosis of LNCaP prostate cancer cells. *The Journal of Biological Chemistry*.

[32] Dai R., Xia Y., Mao L., Mei Y., Xue Y., Hu B. (2012). Involvement of PI3K/Akt pathway in the neuroprotective effect of Sonic hedgehog on cortical neurons under oxidative stress. *Journal of Huazhong University of Science and Technology. Medical Sciences*.

[33] Kim M. Y., Lee J. U., Kim J. H. (2014). Decrease of PKB/Akt phosphorylation is partially mediated by SAPK/JNK activation in serum-free L6 myoblasts starved with low glucose. *Journal of Physical Therapy Science*.

[34] Arboleda G., Morales L. C., Benítez B., Arboleda H. (2009). Regulation of ceramide-induced neuronal death: cell metabolism meets neurodegeneration. *Brain Research Reviews*.

[35] Chen L., Ren F., Zhang H. (2012). Inhibition of glycogen synthase kinase 3beta ameliorates D-GalN/LPS-induced liver injury by reducing endoplasmic reticulum stress-triggered apoptosis. *PLoS ONE*.

[36] Lee W. S., Yi S. M., Yun J. W. (2014). Polyphenols isolated from *Allium cepa* L. induces apoptosis by induction of p53 and suppression of Bcl-2 through inhibiting PI3K/Akt signaling pathway in AGS human cancer cells. *Journal of Cancer Prevention*.

[37] McCormick F. (2004). Cancer: survival pathways meet their end. *Nature*.

[38] Shore G. C., Nguyen M. (2008). Bcl-2 proteins and apoptosis: choose your partner. *Cell*.

[39] Skommer J., Wlodkowic D., Deptala A. (2007). Larger than life: mitochondria and the Bcl-2 family. *Leukemia Research*.

[40] D'Amelio M., Sheng M., Cecconi F. (2012). Caspase-3 in the central nervous system: beyond apoptosis. *Trends in Neurosciences*.

[41] Algeciras-Schimnich A., Barnhart B. C., Peter M. E. (2002). Apoptosis-independent functions of killer caspases. *Current Opinion in Cell Biology*.

[42] Zheng J. H., Viacava Follis A., Kriwacki R. W., Moldoveanu T. (2015). Discoveries and controversies in BCL-2 protein-mediated apoptosis. *The FEBS Journal*.

[43] Vesely D. L., Hoffman B., Liebermann D. A. (2007). Phosphatidylinositol 3-kinase/Akt signaling mediates interleukin-6 protection against p53-induced apoptosis in M1 myeloid leukemic cells. *Oncogene*.

[44] Rasul A., Ding C., Li X. (2012). Dracorhodin perchlorate inhibits PI3K/Akt and NF-*κ*B activation, up-regulates the expression of p53, and enhances apoptosis. *Apoptosis*.

[45] Yang L., Xu L. Z., Liu Z. Q. (2015). Interleukin-13 interferes with activation-induced t-cell apoptosis by repressing p53 expression. *Cellular & Molecular Immunology*.

[46] Henshall D. C., Araki T., Schindler C. K. (2002). Activation of Bcl-2-associated death protein and counterresponse of Akt within cell populations during seizure-induced neuronal death. *The Journal of Neuroscience*.

[47] Yuan L., Wei S., Wang J., Liu X. (2014). Isoorientin induces apoptosis and autophagy simultaneously by reactive oxygen species (ROS)-Related p53, PI3K/Akt, JNK, and p38 signaling pathways in HepG2 cancer cells. *Journal of Agricultural and Food Chemistry*.

[48] Seo B. R., Min K.-J., Cho I. J., Kim S. C., Kwon T. K. (2014). Curcumin significantly enhances dual PI3K/Akt and mTOR inhibitor NVP-BEZ235-induced apoptosis in human renal carcinoma Caki cells through down-regulation of p53-dependent Bcl-2 expression and inhibition of Mcl-1 protein stability. *PLoS ONE*.

